# Metropolitan Hospital Sunday Fund

**Published:** 1897-08-14

**Authors:** 


### METROPOLITAN HOSPITAL SUNDAY FUND.

A Council meeting of the Metropolitan Hospital Sunday Fund was held at the Mansion House on Thursday, 5th inst., for the purpose of receiving the report of the Committee of Distribution (a copy of which will be found on page 344. Sir Sydney H. Waterlow (vice-president) took the chair, in the absence of the Lord Mayor, and there were also present: Sir Dyce Duckworth, Sir Henry Burdett, Sir Edmund Hay Currie, Mr. A. G. Sandeman, the Hon. Sydney Holland, Canon Fleming, Canon Rhodes-Bristowe, the Rev. A. A. Harland, Dr. Hare, Dr. Glover, Messrs. A. Willett, R. Grey, Herman Hoskier, James Christy, G. Drummond, J. Astley Bloxam, F. H. Norman, and Captain Cundy.

The Chairman, in moving that the report of the Committee of Distribution for the year 1897 be approved, and that the several awards recommended be paid as soon as possible, reminded the meeting that that was the Silver Jubilee of the Fund. Twenty-five years ago, at the Mansion House, that Fund was first started, and he thought the work which it had done since then had been most satisfactory. (Hear, hear.) During those 25 years £913,482 had been collected—(hear, hear)—£853,013 had been distributed in awards to different hospitals, and £30,138 had been expended in providing surgical appliances. In the latter connection, assuming one appliance only to each applicant, the Fund had prevented the necessity of 35,802 persons running about all over London trying to collect letters in order to enable them to get surgical appliances. The Distribution Committee's report recommended that £39,705 15s. 6d. should be paid to 132 hospitals and 56 dispensaries. This amount was not quite as large as last year's, when £41,829 was distributed; but when the difficulties under which Hospital Sunday had been worked this year, and the extent to which the public had responded in the shape of subscriptions to hospitals in other directions were considered, the difference was not a very large one. This year the committee proposed to carry forward even less than in previous years. As the members of the Council knew, the Distribution Committee had never, and he hoped would never, propose to pile up anything like a reserve fund; in fact, they had adopted the principle that the money which a generous public was kind enough to place in their hands in any one year was intended for the relief of disease and sickness in the year in which it was given. (Hear, hear.) The work of the Distribution Committee, too, had been very much heavier this year than in the past, the committee having felt that they should act even more stringently on the rules laid down for their guidance, and where they had the least doubt as to the manner in which the funds of a hospital had been expended, or where there appeared to be anything like extravagant management, they had asked for deputations, with the result that 38 hospitals were invited to send representatives to confer with the committee. Of these, 30 sent such deputations, and these conferences were found to be both useful and mutually instructive.

Sir Edmund Hay Currie, in seconding, asked the Distribution Committee to note that, out of the £40,000 they were voting that day, only about £4,000 went to the south of London.

Sir Dyce Duckworth asked why St. Thomas's Hospital, which did apply for a grant, had not received one?

The Chairman said that when St. Thomas's Hospital wrote to say they would like to have a share of the Hospital Sunday Fund they were asked to produce their accounts. Those accounts had not been sent in, and therefore the Committee of Distribution had been unable to do anything more in the matter. He might also mention that a conference had been held at the Mansion House, the Lord Mayor presiding, at which the treasurer of St. Thomas's and the treasurer of Guy's Hospital were present. After a long discussion the treasurer of Guy's went away, he (the chairman) thought he might say satisfied, and the treasurer of St. Thomas's left the Distribution Committee, under the impression that next year he would put his hospital in a position to enable him to apply for a grant. The friends of St. Thomas's might rest assured that the Distribution Committee would carefully consider their application as soon as they were placed in a position to do so. The principle the committee worked on was to give the money where it was most wanted and where it would best serve the end and object for which the Fund was formed, namely, to give the largest amount of relief possible from the sum collected.

Dr. Glover said that last year the question of the abuse of the out-patient department was gone into at the meeting of the Council, and was referred to the consideration of the Distribution Committee. Up to the present time no formal answer or report had been received from the committee, and he would like to know what had been done in the matter. As showing how important the question was he said that, according to the statistics issued by the Fund, although some of the hospitals showed decreases, yet the number of out-patients in twelve or thirteen of the large and important London hospitals showed an increase of some 20,000 over the previous year. Speaking from memory, there were increases of some thousands at the Great Northern Central, Guy's, the London, the North-West London, the Middlesex, University College, West Ham, and Westminster Hospitals.

The Chairman said that Dr. Glover knew what a very difficult question that question of the out-patients was, and that it had to be looked at from five or six different points of view. There were the views of the medical men on the advantage of the out-patient department to the medical school; the views of the clergy with regard to the great advantage which free treatment was to the poor, and how it was appreciated by them; the views of the persons promoting those very admirable institutions the provident dispensaries, and the question of the extent to which those attending provident dispensaries could be increased and the attendance at the out-patient departments decreased. All these views, and others, had to be faced, and the committee had had the question under consideration on several occasions. At the last meeting on the subject, held in July, it was resolved unanimously that the consideration of the special report on the out-patient departments of hospitals be adjourned until the committee have had the opportunity of obtaining more expert evidence, and that a meeting be summoned early in October for that purpose. It would be futile for the committee to bring up a report which did not more or less cover that important and interesting subject.

Sir Henry Burdett, in supporting the adoption of the report, said that, if read carefully and with understanding, it showed that the work done by the committee during the past year had been harder and probably even more thorough than in any previous year of the Fund's existence. He wished to emphasise that fact, because the members of the Council were immensely indebted to the Distribution Committee for the work which they undertook, and which they did so thoroughly and so well. The best evidence of that thoroughness was to be found in the fact that, although it was this committee which really distributed the Fund, there was almost no criticism—at any rate no criticism of an important character—in regard to its work, a fact which was the more remarkable when it was remembered that wherever there was money to be given away, no matter who gave it away, there were always people ready to come forward and say they knew a better way of distribution. (Laughter.) As helping the Council to realise what was meant by the work this year, he said that the fact that 30 deputations had been received (allowing only one hour for each deputation) meant that 30 hours of work were given by the committee to that one matter alone—a very substantial gift to the Fund by some of the busiest men in the City of London. Among the criticisms of the Fund which he had seen it had been said that it took too little account of the needs and far too much of the proportion of the management expenses of the hospitals. As an answer to that, if they looked at the awards they would find that the grants to the Chelsea Hospital for Women and The Hospital for Women, Soho Square, were the same, viz., £306 5s., while the expenses of management at the Chelsea Hospital were 14½ per cent., and at The Hospital for Women only 12¾ per cent. That showed that the needs and merits of all the institutions were carefully gone into. There was one award he would like to have some explanation of. He noticed that one of the best administered and the best found of the London hospitals—the National Hospital for the Paralysed and Epileptic—was to receive a grant of £752 10s., whereas Charing Cross Hospital was to receive £1,006, and the Royal Free Hospital £831. He had selected these three instances because the hospitals had about the same number of beds—the National Hospital, 180 beds; Charing Cross, 175; and the Royal Free, 170. He did not for one moment question the awards in any case, but an explanation in this case as to the special reasons for the differences would give an insight into the way the awards were made, and so would be helpful to many people.

The Chairman explained that the differences were caused by the fact that, as the National Hospital for the Paralysed had during the past three years received an average of £2,000 from patients' payments, the committee did not think it was as much in need of help as Charing Cross, which only received £82 from that source, or the Royal Free, which was a free hospital and received nothing.

The Hon. Sydney Holland asked if they were to understand from that that if a hospital got money from its patients it would receive less from the Hospital Sunday Fund? Did the chairman mean that if a hospital ran into debt its needs were greater than those of a hospital which was thriftily managed, and therefore the Sunday Fund should step in and help the former?

The Chairman said that the committee first examined the management of a hospital, and if they came to the conclusion that its management was thrifty, careful, and efficient, they then considered how much money was required from the public to help the money received from other sources. If a large amount was received from invested property or from patients' payments, less was required from the public.

The resolution was then put, and carried unanimously.

Canon Fleming moved:

"That the cordial thanks of the Council be and are hereby given to Sir Sydney H. Waterlow, Bart. (chairman), and to the other members of the Committee of Distribution, for the special care they have taken this year by the interviewing of numerous deputations, and for the considerable attention bestowed in the preparation of the awards they have recommended."

In doing so he said he felt sure that if there was the slightest feeling on the part of the public that the distribution of the money was not carried out on the very best basis possible, the clergy would be sure to hear of it.

Mr. J. Astley Bloxam seconded, and the motion was carried.

The Chairman, in returning thanks, hoped he would live to see the amount collected by the Fund reach a million.

Canon Bristow proposed, and Dr. Hare seconded, a vote of thanks to the editors of newspapers who had pleaded the needs of hospitals and advocated the cause of the Fund, with a view to increasing the year's collection. The resolution was carried.

Mr. A. G. Sandeman proposed, and Sir Dyce Duckworth seconded, a vote of thanks to the Lord Mayor, who, as president and treasurer, had devoted his valuable time and considerable energy to continue the well-being of the Fund. This was also carried unanimously.

On the motion of Dr. Glover, seconded by Sir Henry Burdett, a vote of thanks was accorded the chairman, and the proceedings terminated.

### PARTICULARS OF THE AWARDS.

The following is the report of the Committee of Distribution adopted by the Council on the 5th inst:—

"Your committee have now to report their recommendation of awards to 188 institutions enumerated in the appendix attached hereto, being an increase of 83 since the first awards were made in 1873, and 8 more than last year. The total amount available for distribution, after allowing for liabilities and the usual current expenses, is about £40,000. Of this total £39,705 15s. 6d. is now recommended to be paid to 132 hospitals and 56 dispensaries. Five per cent. of the total sum collected, viz., about £2,000, is set apart to purchase surgical appliances (in obedience to Law XII.), in monthly proportions during the ensuing year. Your committee recommend that all payments to the Fund after this date be carried to the credit of next year's Fund.

"In compliance with an order of the Council, and for the special use of its members, tables of statistics have been prepared as usual, showing an analysis of the number of beds in hospitals, the cost of patients both at hospitals and dispensaries, the proportionate expense of management, as well as other valuable information, and copies are sent to every member of the Council.

"The amount available for distribution is, as was naturally expected, rather less than the amount available last year, but your committee feel that it is as large a sum as could be anticipated, having regard to the circumstances of the Jubilee Year, in which so strong an appeal has been made in other directions and responded to by the public in favour of our metropolitan hospitals.

"For the last twenty-five years the public have nobly responded to the earnest appeal made by ministers of religion in nearly 2,000 places of worship on Hospital Sunday. Your committee are of opinion that the amount received this year fully justifies them in the conclusion that the confidence of the public in the Metropolitan Hospital Sunday Fund has in no degree abated, and that the collections in future years, which it is to be hoped will be made under ordinary circumstances, will continue to increase from time to time, as they have done in past years. Appeals have been made from year to year mainly on the ground that it is a religious duty for everyone to give what they can towards the relief and assistance of the sick poor of this great metropolis who are unable to provide such help for themselves, and your committee fully believe that an appeal on these grounds will never be made in vain.

"In order to arrive as accurately as possible at the needs and merits of the 188 institutions making applications for a grant from the funds at your disposal, your committee have deemed it necessary to invite the attendance of a deputation from the governing bodies of a much larger number of institutions than in former years. They invited representatives from 38 institutions. In 30 cases deputations were sent to confer with your committee; four of the institutions expressed their willingness to leave themselves in the hands of your committee, and four others took no notice of the invitation. The conferences with the 30 deputations were found to be both useful and mutually instructive. In some cases the objections were removed or modified by the explanations given; in other cases the explanations at the conference only confirmed the impressions your committee had formed.

"The work was much facilitated by the fact that the accounts are universally rendered on the uniform system."

### Twenty-seven General Hospitals.

Charing Cross Hospital, £1,006 5s.; French Hospital, £323 15s.; German Hospital, £393 15s.; Great Northern Central Hospital, £481 5s.; Guy's Hospital, £1,312 10s.; Hampstead Hospital, £131 5s.; Italian Hospital, £61 5s.; King's College Hospital, £1,400; London Hospital, £3,937 10s.; London Homœopathic Hospital, £245; Phillips Memorial Homœopathic Hospital, £30 12s. 6d.; London Temperance Hospital, £647 10s.; Metropolitan Hospital, £481 5s.; Mildmay Mission Hospital, £262 10s.; Miller Hospital and Royal Kent Dispensary, £236 5s.; North-West London Hospital, £350; Poplar Hospital, £402 10s.; Royal Free Hospital, £831 5s.; St. George's Hospital, £1,356 5s.; SS. John and Elizabeth Hospital, £87 10s.; St. Mary's Hospital, £1,706 5s.; Seamen's Hospital Society, £918 15s.: The Middlesex Hospital, £1,925; University College Hospital, £1,181 5s.; West Ham Hospital, £280; West London Hospital, £525; Westminster Hospital, £1,093 15s.

### Special Hospitals.

Five Chest Hospitals.—City of London Hospital for Diseases of the Chest, Victoria Park, £927 10s.; Hospital for Consumption, Brompton, £1,391 5s.; North London Consumption Hospital, Hampstead, £262 10s.; Royal Hospital for Diseases of the Chest, City Road, £350; Royal National Hospital for Consumption, Ventnor, £262 10s.

Fourteen Children's Hospitals.—Alexandra Hospital for Hip Disease, W.C., £148 15s.; Barnet Home Hospital, £35; Belgrave Hospital for Children, S.W., £122 10s.; Cheyne Hospital for Incurable Children, S.W., £122 10s.; East London Hospital for Children, Shadwell, E., £393 15s.; Evelina Hospital for Sick Children, Southwark, S.E., £393 15s.; Home for Incurable Children, Maida Vale, W., £61 5s.; Home for Sick Children, Sydenham, S.E., £122 10s.; Hospital for Sick Children, Great Ormond Street, W.C., £700; North-Eastern Hospital for Children, Hackney Road, N.E., £262 10s.; Paddington Green Hospital for Children, W., £148 15s.; Victoria Hospital for Children, Queen's Road, Chelsea, S.W., £481 5s.; Victoria Home, Margate, £26 5s.; "The Vine," Sevenoaks, £26 5s.

Six Lying-in Hospitals.—British Lying-in Hospital, Endell Street, W.C., £26 5s.; City of London Lying-in Hospital, City Road, E.C., £65 12s. 6d.; Clapham Maternity Hospital, £30 12s. 6d.; East End Mothers' Home, £61 5s.; General Lying-in Hospital, Lambeth, S.E., £52 10s.; Queen Charlotte's Lying-in Hospital, Marylebone Road, W., £201 5s.

Six Hospitals for Women.—Chelsea Hospital for Women, S.W., £306 5s.; Hospital for Women, Soho Square, W., £306 5s.; Grosvenor Hospital for Women and Children, Vincent Square, S.W., £43 15s.; New Hospital for Women and Children, Euston Road, W.C., £192 10s.; Royal Hospital for Children and Women, Lambeth, S.E., £218 15s.; Samaritan Free Hospital, Lower Seymour Street, W., £490.

### Twenty-seven Other Special Hospitals.

Cancer Hospital, Brompton, S.W., £568 15s.; London Fever Hospital, Islington, N., £875; Gordon Hospital for Fistula, Vauxhall Bridge Road, S.W., £36 15s.; St. Mark's Hospital for Fistula, City Road, E.C., £52 10s.; National Hospital for Diseases of the Heart and Paralysis, Soho Square, W., £52 10s.; Female Lock Hospital, Harrow Road, W., £122 10s.; Hospital for Epilepsy, Paralysis, and other Diseases of the Nervous System, Regent's Park, N.W., £56 17s. 6d.; National Hospital for the Paralysed and Epileptic, W.C., £752 10s.; West End Hospital for Diseases of the Nervous System, £96 5s.; Central London Ophthalmic Hospital, Gray's Inn Road, W.C., £26 5s.; Royal Eye Hospital, St. George's Circus, S.E., £166 5s.; Royal London Ophthalmic Hospital, Moorfields, E.C., £490; Royal Westminster Ophthalmic Hospital, Charing Cross, W.C., £87 10s.; Western Ophthalmic Hospital, Marylebone Road, W., £39 7s. 6d.; City Orthopædic Hospital, Hatton Garden, E.C., £65 12s. 6d.; National Orthopædic Hospital, Great Portland Street, W., £70; Royal Orthopædic Hospital, Oxford Street, W., £61 5s.; Royal Sea-Bathing Infirmary, Margate, £297 10s.; Hospital for Diseases of the Skin, Stamford Street, S.E., nil; Western Skin Hospital nil; St. Peter's Hospital for Stone, Covent Garden, £26 5s.; Central London Throat and Ear Hospital, Gray's Inn Road, W.C., £35; Hospital for Diseases of the Throat, Golden Square, W.C., nil; Metropolitan Ear and Nose Hospital, nil; Royal Ear Hospital, Frith Street, W., £7 17s. 6d.; Dental Hospital of London, Leicester Square, W.C., £109 7s. 6d.; National Dental Hospital, 149, Great Portland Street, W., £105.

### Twenty-seven Convalescent Hospitals.

Metropolitan Convalescent Institution, Sackville Street, W., £568 15s.; Bexhill Convalescent Home, Sackville Street, W., £262 10s.; All Saints' Convalescent Hospital, Eastbourne, £481 5s.; Mrs. Gladstone's Convalescent Home, Woodford, £87 10s.; Hahnemann Convalescent Home, Bournemouth, £35; Hanwell Convalescent Home, £13 2s. 6d.; Herbert Convalescent Home, Bournemouth, £17 10s.; Herne Bay Baldwin-Brown Convalescent Home, £21 17s. 6d.; Herne Bay Mothers' Home, £39 7s. 6d.; Homœopathic Convalescent Home, Eastbourne, £13 2s. 6d.; King's College Hospital Convalescent Home, Hemel Hempstead, £56 17s. 6d.; Mrs. Kitto's Convalescent Home, Reigate, £78 15s.; London and Brighton Female Convalescent Home, £26 5s.; Mary Wardell Convalescent Home for Scarlet Fever, £87 10s.; Morley House Convalescent Home for Working Men, £96 5s.; St. Andrew's Convalescent Hospital, Clewer, £157 10s.; St. Andrew's Convalescent Home, Folkestone, £245; St. John's Home for Convalescent and Crippled Children, Brighton, £70; St. Joseph's Convalescent Home, Bournemouth, £48 2s. 6d.; St. Leonard's-on-Sea Convalescent Home for Poor Children, £87 10s.; St. Michael's Convalescent Home, Westgate-on-Sea, £26 5s.; Seaside Convalescent Hospital, Seaford, £122 10s.; Warley Convalescent Cottage Hospital, Essex, £6 2s. 6d.; Ascot Priory Convalescent Home, £61 5s.; Friendly Societies' Convalescent Home, Dover, £30 12s. 6d.; Police Seaside Home, Brighton, £17 10s.; St. Mary's Convalescent Home, Shortlands, £13 2s. 6d.

### Thirteen Cottage Hospitals.

Beckenham Cottage Hospital, £43 15s.; Blackheath and Charlton Cottage Hospital, £61 5s.; Bromley, Kent, Cottage Hospital, £72 12s. 6d.; Chislehurst, Sidcup, and Cray Valley Cottage Hospital, £39 7s. 6d.; Eltham Cottage Hospital, £28 17s. 6d.; Enfield Cottage Hospital, £39 7s. 6d.; Epsom and Ewell Cottage Hospital, £61 5s.; Hounslow Cottage Hospital, £17 10s.; Mildmay Cottage Hospital, £61 5s.; Reigate and Redhill Cottage Hospital, £52 10s.; Sidcup Cottage Hospital, £30 12s. 6d.; Wimbledon Cottage Hospital, £35; Woolwich and Plumstead Cottage Hospital, £30 12s. 6d.

### Seven Institutions.

Establishment for Gentlewomen, Harley Street, £105; St. Saviour's Hospital and Nursing Home, £105; National Sanatorium for Consumption, Bournemouth, £52 10s.; Invalid Asylum, Stoke Newington, £52 10s.; Firs Home, Bournemouth, £35; St. Catherine's Home, Ventnor, £26 5s.; Friedenheim Home for the Dying, £218 15s.

### Fifty-six Dispensaries.

Battersea Provident Dispensary, £65 12s. 6d.; Bloomsbury Provident Dispensary, £10 10s.; Brixton, &c., Dispensary, £52 10s.; Brompton Provident Dispensary, £21 17s. 6d.; Camberwell Provident Dispensary, £87 10s.; Camden Provident Dispensary, £11 7s. 6d.; Chelsea, Brompton, and Belgrave Dispensary, £42; Chelsea Provident Dispensary, £13 2s. 6d.; Child's Hill Provident Dispensary, £8 15s.; City Dispensary, £61 5s.; City of London and East London Dispensary, £21; Clapham General and Provident Dispensary, £22 15s.; Deptford Medical Mission, £14; Eastern Dispensary, £28 17s. 6d.; East Dulwich Provident Dispensary,

£13 2s. 6d.; Farringdon General Dispensary, £35; Finsbury Dispensary, £26 5s.; Forest Hill Dispensary, £26 5s.; Gipsy Hill Provident Dispensary, £4 7s. 6d.; Hackney Provident Dispensary, £6 2s. 6d.; Hampstead Provident Dispensary, £39 7s. 6d.; Holloway and North Islington Dispensary, £56 17s. 6d.; Islington Dispensary, £39 7s. 6d.; Kensal Town Provident Dispensary, £4 7s. 6d.; Kensington Dispensary, £61 5s.; Kilburn, Maida Vale, and St. John's Wood Dispensary, £28; Kilburn Provident Medical Institution, £26 5s.; London Dispensary, £13 2s. 6d.; London Medical Mission, Endell Street, £52 10s.; Metropolitan Dispensary, £43 15s.; Notting Hill Dispensary and Maternity (Provident), £13 2s. 6d.; Paddington Provident Dispensary, £28; Pimlico Provident Dispensary (Lupus Street), £14; Portland Town Dispensary, £14; Portobello Road Provident Dispensary, £5 5s.; Public Dispensary, £39 7s. 6d.; Queen Adelaide's Dispensary, £19 5s.; Royal General Dispensary, £35; Royal Pimlico Provident Dispensary, £43 15s.; Royal South London Dispensary, £43 15s.; St. George's and St. James's Dispensary, £39 7s. 6d.; St. George's, Hanover Square, Provident Dispensary, £35; St. John's Wood and Portland Town Provident Dispensary, £32 7s. 6d.; St. Marylebone General Dispensary, £43 15s.; St. Pancras and Northern Dispensary, £39 7s. 6d.: South Lambeth, Stockwell, and North Brixton Dispensary, £39 7s. 6d.; Stamford Hill, Stoke Newington, &c., Dispensary, £43 15s.; Tower Hamlets Dispensary, £31 10s.; Walworth Provident Dispensary, £9 12s. 6d.; Wandsworth Common Provident Dispensary, £7 17s. 6d.; Westbourne Provident Dispensary and Maternity, £12 5s.; Western Dispensary, £26 5s.; Western General Dispensary, £118 2s. 6d.; Westminster General Dispensary, £26 5s.; Whitechapel Provident Dispensary, £16 12s. 6d.; Woolwich Provident Dispensary £8 15s.

